# Intrathymic Selection and Defects in the Thymic Epithelial Cell Development

**DOI:** 10.3390/cells9102226

**Published:** 2020-10-02

**Authors:** Javier García-Ceca, Sara Montero-Herradón, Agustín G. Zapata

**Affiliations:** 1 Department of Cell Biology, Faculty of Biology, Complutense University of Madrid, 28040 Madrid, Spain; jgarciaceca@bio.ucm.es (J.G.-C.); saramontero@ucm.es (S.M.-H.); 2 Health Research Institute, Hospital 12 de Octubre (imas12), 28041 Madrid, Spain

**Keywords:** thymic epithelial cells, thymocyte education, regulatory T-cells, Eph/ephrins

## Abstract

Intimate interactions between thymic epithelial cells (TECs) and thymocytes (T) have been repeatedly reported as essential for performing intrathymic T-cell education. Nevertheless, it has been described that animals exhibiting defects in these interactions were capable of a proper positive and negative T-cell selection. In the current review, we first examined distinct types of TECs and their possible role in the immune surveillance. However, EphB-deficient thymi that exhibit profound thymic epithelial (TE) alterations do not exhibit important immunological defects. Eph and their ligands, the ephrins, are implicated in cell attachment/detachment and govern, therefore, TEC–T interactions. On this basis, we hypothesized that a few normal TE areas could be enough for a proper phenotypical and functional maturation of T lymphocytes. Then, we evaluated in vivo how many TECs would be necessary for supporting a normal T-cell differentiation, concluding that a significantly low number of TEC are still capable of supporting normal T lymphocyte maturation, whereas with fewer numbers, T-cell maturation is not possible.

## 1. Introduction

The immune system has developed several strategies to properly control the immune responses. These strategies allow peripheral T lymphocytes to respond strongly to non-self-antigen determinants presented by self-MHC molecules, whereas they do not elicit a response when confronted to self-antigens, avoiding the appearance of autoimmune syndromes. Central to this behavior is the positive selection of thymocytes in the thymic cortex and the removal of autoreactive T-cell clones (negative selection) in the medulla [1,2], as well as the generation of the so-called T regulatory (Treg) cells [3].

The thymus is a primary lymphoid organ derived from the endoderm of the 3rd pharyngeal pouch implicated in the maturation of thymocytes and, therefore, key for establishing the immune surveillance. This organ does not contain self-renewing lymphoid precursor cells and therefore, is colonized by lymphoid precursors coming either from the fetal liver through mesenchyme or from the adult bone marrow via the blood vessels [4]. Within the thymus, the developing thymocytes move throughout a 3D thymic epithelial (TE) network, interacting with the thymic epithelial cells (TECs) of two histologically different compartments: the cortex and the medulla [5].

Both DN (CD4^−^CD8^−^) thymocytes, derived directly from the lymphoid precursor cells seeding the thymus and DP (CD4^+^CD8^+^) thymocytes, migrate throughout the cortical (c) epithelium. During this journey, cTEC present self-peptides with the concourse of MHC molecules to DP cells (see later). Those thymocytes whose TCRαβ interact with peptide-MHC complexes with intermediate affinity/avidity will be positively selected and will differentiate to mature SP (CD4^+^CD8^−^ or CD4^−^CD8^+^) cells [5]. Other DP thymocytes that do not recognize any MHC–peptide complexes will die by neglect [6].

Positively selected SP thymocytes up-regulate the expression of CCR7 and migrate into the medulla through the cortico-medullary junction to interact with medullary (m) TECs that express ligands of CCR7 (i.e., CCL19, CCL21) [7]. mTECs have the capacity to present peptides derived from tissue proteins (TSA, tissue specific antigens) expressed outside the thymus with the concourse of, at least, two different transcription factors: Autoimmune regulator (Aire) [5,8,9,10,11] and Forebrain embryonic Zn-finger-like (Fefz2) [12]. These mTEC–thymocyte interactions are essential for the induction of thymic selection, as thymocytes whose TCRαβ recognize with high-affinity TSA are eliminated by apoptosis (negative selection) [5,10] or differentiate to Treg cells [3].

In the next sections, we will analyze in detail these processes and will describe some experimental models in which, despite profound alterations of the thymic epithelium, the intrathymic T-cell education seems to proceed properly. Finally, we will propose some possible explanations to this behavior.

## 2. Thymic Epithelium Is Originated from Epithelial Progenitors (TEPCs) whose Nature Is Controversial

As mentioned above, TE derives from the endoderm of the 3rd pharyngeal pouch [4]. In this early thymic primordium, a common bipotent thymic progenitor cell was identified capable of giving rise to both committed cTEC precursors and mTE progenitor cells [13,14,15,16,17,18]. However, the phenotype of these cells is a matter of discussion and other studies have reported the existence of lineage-specific progenitor cells either for cTECs or mTECs [19,20]. In this respect, it is important to remark that embryonic thymic epithelial progenitor cells (TEPCs) express cell markers (i.e., β5t, CD205, IL7) specific of the cTEC lineage of adult thymus [21,22,23]. These results supported a model of serial progression for explaining the establishment of two major TEC subsets, cortical and medullary. Recently, single cell-RNA sequencing analysis [24] confirmed that most embryonic TECs show a strong cTEC footprint. According to this model, TEPCs would be committed to the cortical epithelial cell lineage by default, whereas the differentiation into mTECs would require the activation of a medullary specific transcriptional program together with the downregulation of cTEC committed genes [10].

On the other hand, embryonic and adult TEPCs seem to be different. Some studies have reported that adult thymi do not contain TEPCs with characteristics of cTECs [19,20] and others remark that bipotent TEPCs do exit in the adult thymus but exhibiting their own molecular characteristics [13,14,15,25]. In any case, adult TEPCs apparently lose their embryonic features, whereas committed progenitor cells either with cTEC or mTEC lineages appear [15,19,20,24,26].

## 3. Cortical Epithelium and Positive Selection

Reported cTEC specific cell markers are few (i.e., K8, Ly51, CD205) and, consequently, it is difficult to establish conclusively both the phenotype and function of cTEC subsets. The existence of bipotent TEPCs has been reported in a small subset of CD45^−^EpCAM^+^Ly51^lo^UEA1^−/lo^MHCII^lo^Intα6^hi^Sca-1^hi^ TECs [14]. This epithelial cell population is radioresistant and proliferates during thymic regeneration [27]. Previously, Shakib et al. [28] had described a CD205^+^CD40^−^ cTEC progenitor population enriched in proliferating cells that expressed lower levels of cTEC-specific transcripts (i.e., β5t and cathepsin L), than the more mature cTECs. In addition, a small subset of CD205^+^ TECs has been reported as immature cTECs [21].

The cortical epithelium is involved in the maturation of DN thymocytes into DP cells through the production of IL7 [29,30] and the surface expression of several Notch ligands, largely Delta-like 4 (Dll4) [31,32], and presents MHC-peptides to DP thymocytes, an essential process for their positive selection. However, it is unclear which cTEC subset is specifically involved in these functions. In adult thymic cortex, cTEC subsets can be immunohistochemically distinguished according to their levels of Ly51 expression. Ly51^hi^ cTECs occupy both the cortico-medullary border and the subcapsule, whereas Ly51^lo^ cells occur throughout the cortex. By flow cytometry, Ly51^hi^ cTECs were MHCII^hi^ and the Ly51^lo^ cell subset correlated with the MHCII^lo^ ones [33,34,35]. In addition, adult Ly51^hi^ cTECs express Dll4, whereas most of the Ly51^lo^ cell subset is Dll4^−^ [35,36,37,38]. Accordingly, Ly51^hi^ cTEC might represent a niche for DN cells whereas Ly51^lo^ ones would constitute the niche where DP thymocytes are selected [35].

During their journey throughout thymic cortex, developing thymocytes undergo selection twice. DN3 (CD4^−^CD8^−^CD44^−^CD25^+^) cells rearrange the TCRαβ alleles [39]; those successfully arranged TCRβ chains are associated with an invariant pre-Tα chain constituting a pre-TCR that apparently does not recognize any ligands [40], but presumably oligomerizes [41], and signals resulting in important proliferation and progression to the DP cell compartment through DN4 (CD4^−^CD8^−^CD44^−^CD25^−^) cells and an intermediate, immature SP (CD4^+^CD8^−^ or CD4^−^CD8^+^) stage [39,42]. Then, DP thymocytes that have rearranged mature TCRαβ receptors are again selected by MHC-peptide complexes presented by cTECs, which have special mechanisms for processing these peptides. In the case of MHCI-peptide complexes, cTECs have thymic proteasomes that contain the β5t protein encoded by Psmb11 gene [43]. Apparently, the β5t protein of thymoproteasomes has lower chymotrypsin activity than the subunits of the immunoproteasomes consequently producing different peptides inside than outside the thymus. Intimate relationship between thymoproteosome activity and positive selection has been demonstrated in Psmb11^−/−^ mice that exhibit significantly reduced proportions of CD4^−^CD8^+^ SP thymocytes and the remaining cells are functionally compromised showing an altered TCRαβ repertoire [44,45]. In addition, a single-nucleotide polymorphism that produces a thymoproteosome variant (G49s) is associated with the appearance of Sjögren’s syndrome and impairs positive selection of CD4^−^CD8^+^ thymocytes [2].

Similarly, the generation of a proper repertoire of selecting MHCII-associated self-peptides depends on the presence of both a thymus-specific serine protease, TSSP and the lysosomal protease, cathepsin L in cTECs. Accordingly, alterations in any of these proteases impair the positive selection of CD4^+^CD8^−^ SP cells [46,47].

## 4. mTECs Constitute A Heterogeneous Thymic Cell Population Involved in both Negative Selection and Treg Cell Generation

The medulla displays an important array of mTEC subsets whose functions in the thymocyte education has been extensively studied [11,48]. Currently, it is assumed the existence of immature mTECs, mature mTECs implicated in negative selection and the production of Treg cells, and a post mature stage whose functional significance is more controversial.

In addition, mTEPCs seem to be different in embryonic and adult mice. Whereas the former ones include Claudin (Cld)3/4^hi^ progenitor cells, the expression of this cell marker disappears in the postnatal TEPCs, and Cld3/4^hi^ cells seem to be restricted to mature Aire^+^ and terminally differentiated mTECs [49]. On the other hand, previous reports classified mTECs into immature MHCII^lo^CD80^lo^ cells (mTEC^lo^ or mTEC-I) and mature MHCII^hi^CD80^hi^ cells (mTEC^hi^ or mTEC-II), but really mTEC^lo^ cells constitute a heterogeneous cell population that could contain both TEPCs and terminal post-Aire cells [26,50,51,52,53,54,55]. Therefore, these mTEC^lo^ cells presumably consist, at least, of two cell subsets: putative adult mTEPCs, that highly express podoplanin and ligands of CCR7, such as CCL21a, CCL21b, CCL21c [56,57] and predominantly occupy the cortico-medullary border [26,58], and a more mature CCL21-producing mTEC subpopulation involved in the recruitment of positively selected CCR7^+^ thymocytes into the medulla [26,57]. Furthermore, Aire^+^ terminally differentiated mTECs downregulate their specific cell markers (i.e., Aire, MHCII, and CD80) giving rise to Aire^−/lo^MHCII^lo^CD80^lo^ cells that could be therefore phenotypically considered immature mTECs.

Apart from the upregulation of other cell markers, such as MHCII and CD80 involved in the antigen presentation by mTECs, the most representative marker of the mature mTEC population is the transcription factor Aire. Mature mTEC^hi^ acquire Aire expression after RANK-RANKL signaling in immature mTECs^lo^. Thus, mice deficient in RANK or RANKL lack Aire^+^ mTECs^hi^ in the embryonic thymus [59,60]. When the deletion of these molecules was tested in neonatal mice, the reduction of mTEC^hi^ cells was not complete [61]. In addition, the CD40-mediated effects on mTEC maturation are weaker that those exerted by RANK signals [60,62].

Firstly, Aire was reported as a defective gene product associated with an autoimmune syndrome called autoimmune polyglandular syndrome type 1 (APS1) [63,64]. The syndrome coursed with autoimmunity affecting diverse organs [65] and hypersensitivity to mucocutaneous candidiasis associated with autoimmune responses against Th17 cytokines, largely IFNα [66,67,68].

Aire promotes the expression of thousands of TSAs that govern the deletion of autoreactive T-cells [8,69,70,71] and the generation of self-reactive Treg cells [72,73,74]. However, Aire is only expressed in 30% of mTECs and controls the expression of approximately 40% of TSAs [12], but the total pool of TSAs expressed in mTECs is highly diverse covering most of the putative self-antigens [75,76]. These results suggest that other cells and/or molecules would be implicated in the central tolerance (see below). In fact, the expression of Aire-dependent TSAs is both stochastic and ordered: only a small fraction of mTECs express a specific TSAs and, at the same time, there is a general pattern of multiple co-expression of scarcely related types of TSAs within the individual mTECs [77,78,79].

Despite the obvious relevance of Aire expression for the establishment of central tolerance, numerous TSAs are expressed in Aire^−/−^ mTECs [77,80]. As mentioned above, another transcription factor, Fezf2, has been recently reported for promoting the expression of a second group of TSAs distinct from the Aire-dependent TSAs [81]. Fezf2-deficient mice do not have defects in positive selection but show altered repertoire of TCR V_β_ in both CD4^+^ and CD8^+^ T-cells. Additionally, specific elimination of Fezf2 gene in TECs results in autoimmunity with presence of tissue lymphocyte infiltrations and auto-antibodies [12]. Apparently, Aire and Fezf2 genes are controlled independently and do not regulate each other, although recent results indicate that RANK/RANKL signals are key for the appearance of both transcription factors [82]. In any case, only 60% of TSA genes are regulated by Aire and/or Fezf2 suggesting that there are still other unknown mechanisms for TSA production in the thymus. In this respect, the remaining source of TSAs could be peripheral DCs proposed to transport peripheral TSAs into the thymus and to induce T-cell deletion and Treg cell development [1,83,84,85,86,87]. In addition, mTECs transport self-antigens to intrathymic DCs in an Aire-dependent process [88,89].

Increasing evidence supports that mTEC differentiation continues beyond the Aire^+^ stages of thymic epithelium development, to give rise to the so-called tuft cells as well as to terminally differentiated epithelial cells that form a part of the named Hassall’s bodies (or corpuscles) [57,90,91]. These terminal epithelial cells express numerous markers of differentiated keratinocytes of the outer epidermal layers, such as keratin 1, keratin 10, involucrins, desmogleins, and Serine protease inhibitor Kazal type 5 (SPINK5) [10]. Whether Aire governs or not this final differentiation of mTECs is a matter of discussion [10]. In addition, the functions of these terminal mTECs are controversial. Although they have lost the MHCII expression making efficient antigen presentation to SP thymocytes difficult, they retain a large fraction of both Aire-dependent and Aire-independent TSA that could transfer to DCs. In addition, human Hassall’s bodies, but not murine ones [92], produce Thymus stromal lymphopoietin (TSLP) that induces Foxp3^+^ Treg cells [93].

Tuft cells are neonatal epithelial cells, firstly identified in mucosae [94,95] and later in the thymus [26,50,96,97]. Studies of lineage tracing demonstrate that more than half of thymic tuft cells derive from mature mTECs^hi^ but the rest do not seem to follow the Aire-expressing cell lineage [26,50]. Furthermore, the genetic signature of thymic tuft cells seems to be unique and, in any case, very different from that of Aire^+^ mTECs.

Mucosal tuft cells produce IL25 and express genes involved in the synthesis of acetylcholine, prostaglandins, or leukotrienes, and in the signaling of taste reception, such as Phospholypase C beta 2 (Plcb2), Transient receptor potential channel subfamily 5 (Trpm5), G protein subunit beta 3 (Gnb3), and G protein subunit gamma 13 (Gng13). Thymic tuft cells also express several members of the taste receptor 2 family as well as L1CAM, DLCK1, MHCII, and CD74, but not other genes characteristic of mucosal tuft cells (i.e., Galectins 2 and 4 (Lgals 2 and Lgals4), Mucin13, Fatty acid binding protein 1 (Fabp1), and Apolipoprotein A1 (Apoa1)) [10].

**Figure 1:**
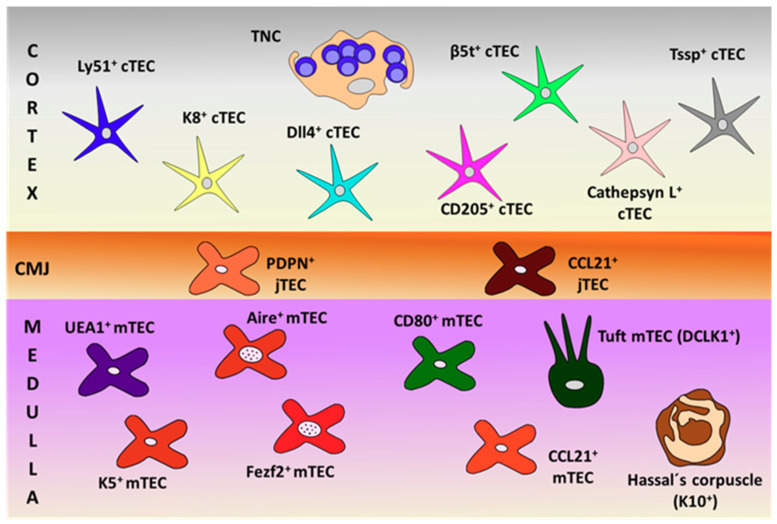
Schematic representation of thymic epithelial cell (TEC) subsets, defined by their specific cell markers in the different thymic compartments (cortex, cortico-medullary junction (CMJ) and medulla). PDPN: podoplanin; TNC: thymic nurse complexes.

On the other hand, the functions of thymic tuft cells are largely unknown. Studies on tuft cell-deficient thymuses grafted in mice immunized with IL25 indicate that tuft cells are key to prevent the generation of anti-IL25 antibodies [50]. However, thymic tuft cells do not appear to be good APC because they weakly express MHCII. In addition, they could be related to other thymic cell subsets because tuft cell deficiency results in reduced numbers of both NKT2 cells and Eomes^+^CD8^+^ SP thymocytes [50]. Figure 1 summarizes all these described TECs.

## 5. The Condition of EphB-Deficient Thymuses

In the previous sections, we have examined the relevance of distinct TEC populations for the positive and negative selection of developing thymocytes. In general terms, the evidence supports that proper TEC-thymocyte interactions are a requisite for an adequate functional education of thymocytes and for the generation of a proper TCR repertoire. However, we [98] and other authors [82,99,100,101] have observed studying different experimental models that thymuses showing profound epithelial alterations maintain normal proportions of thymocyte subsets, including positive and negative selected thymocytes, and Treg cells.

Twenty years ago, we demonstrated the expression of erythropoietin-producing hepatocyte (Eph) tyrosine kinase receptors and their ligands, Eph receptor-interacting proteins (ephrins), in the thymus [102], initiating a series of studies on their role in the organ. Particularly, we demonstrated the relevance of EphB2 and EphB3 and their ligands, ephrin-B1 and ephrin-B2, in the organization of a 3D TE network and the establishment of TEC-thymocyte functional interactions [103].

The Eph kinase receptors and their ligands, the ephrins provide positional information and participate in cell-to-cell contacts, cell migration, and cellular attachment/detachment, as well as in cell survival, differentiation, and organogenesis [104,105]. Eph kinases are subdivided in two classes according their molecular structure and sequence identity: A (10 members) and B (6 members). Preferentially, but not exclusively, EphA interact with ephrins-A (6 members) and EphB with ephrin-B (3 members). Remarkably, Eph/ephrin interactions cause bidirectional signaling with forward signaling through Eph and reverse one in those transmitted via ephrins [106].

EphB-deficient thymuses, although specific in their phenotypes, show some common features, such as hypocellularity, importantly altered epithelium but few changes in the proportions of thymocyte subsets. In addition, the mutant phenotype becomes evident early in the development but gradually increases in correlation with the establishment of TEC-thymocyte interactions [98,103].

Mutant thymuses have lower numbers of both thymocytes [107,108] and TECs [109,110]. Moreover, they are colonized in vitro [111] and in vivo [108,112] by lower numbers of recent lymphoid emigrants that, in addition, mature slower than the WT ones in correlation with a low production of chemokines by mutant TECs [111]. Increased proportions of apoptotic thymocytes and reduced percentages of cycling cells [107] also contribute to the lymphoid hypocellularity of EphB-deficient thymuses. These reduced proportions of dividing thymocytes could be related to diminished production of Dll4 and/or diminished IL7 receptor α chain transcripts [110], a finding also reported in the thymocytes specifically deleted of ephrin-B1 and/or ephrin-B2 genes [113].

This pattern of thymocyte development correlates with a similar behavior of mutant TECs, such as the decreased proportions of immature MTS20^+^ cells reflect [108], their delayed maturation and the reduced total TEC numbers. Moreover, the mutant thymi show reduced proportions of cycling TECs due to both the reduced number of seeding lymphoid progenitors into the thymus [108,110] and lower numbers of transcripts of FGF and its receptor FGFR2iiib [110], both molecules involved in epithelial cell proliferation [100,114]. On the other hand, increased percentages of both fetal and neonatal apoptotic TECs [115] also contribute to the reduced cell content of EphB-deficient thymuses.

The histological organization of EphB-deficient thymuses is also severely altered. Murine WT thymic medulla is formed by small, scattered foci that expand and converge after birth [116], but in adult mutant thymuses, particularly in the EphB2^−/−^ ones, the fusion in a unique medulla is impaired and the small, isolated epithelial islets remain [109]. In support, reaggregate thymus organ cultures (RTOC) established either with EphB2^−/−^ or EphB3^−/−^ thymic lobes or with WT ones treated either with blocking anti-EphB2 or anti-EphB3 antibodies show more and smaller medullary foci as compared with untreated WT RTOCs [117].

In addition, in many thymic areas, thymocytes and TECs are physically isolated because EphB2^−/−^ TECs exhibit shortened cell processes or a lack of them, as in the EphB3^−/−^ ones [109]. Similar phenotypes occur in RTOCs derived from WT thymi treated with blocking anti-EphB2 or anti-EphB3 antibodies [98]. Obviously, these changes together with the abovementioned increased numbers of apoptotic TECs, prevent the proper TEC-thymocyte interactions, essential for thymocyte selection, and favor the appearance of epithelial free areas (EFAs) [118].

On the other hand, the lack of EphB, particularly EphB3, results in delayed cTEC maturation from the earliest stages of development [110]. Thus, E14.5 and E15.5 mutant thymuses accumulate immature Ly51^+^CD205^−^ cTEC, whereas the proportions of mature Ly51^+^CD205^+^ cells decrease. The evolution of other cTEC subsets identified by other cell markers, such as CD40 and MHCII, follows the same pattern. Remarkably, the proportions of β5t^+^ cTECs involved in antigen processing and presentation via MHCI molecules to DP thymocytes is also significantly reduced in mutant embryonic thymuses [110]. On the contrary, the most important alterations of the medulla occur when EphB2 is lacking. These animals show delayed TEC development that courses with accumulation of Cld3,4^hi^SSEA1^+^ TEPCs and, consequently, with a decrease in mature mTECs. Proportions of Aire^+^ cells are also significantly reduced in both mutant postnatal thymuses [117].

Although little is known on the molecules that regulate the thymic cortical epithelium, in the medulla, molecules of the TNF/TNFR family, and particularly the RANK/RANKL pair are important [24,59,60,119] although other members, such as osteoprotegerin [61] and LTβR [120] might modulate their effects [121]. Although in EphB-deficient fetal and postnatal mice we did not see changes in the expression of both RANK and LTβR transcripts, they exhibited decreased proportions of the E15.5 RANKL^+^Vγ5^+^ thymocytes. This reduction and/or just a partial reduction of cell-to-cell interactions between these lymphoid cells and the mTECs could explain the alterations observed in mutant thymi, particularly the reduction of Aire^+^ mTECs, profoundly affected in the absence of RANK/RANKL signaling [60,61]. Indeed, the defective maturation of mutant mTEC is recovered after supply of agonist anti-RANK antibodies for 4 days. Accordingly, the lack of EphB affects the intimate molecular machinery governing mTEC maturation [117].

**Figure 2:**
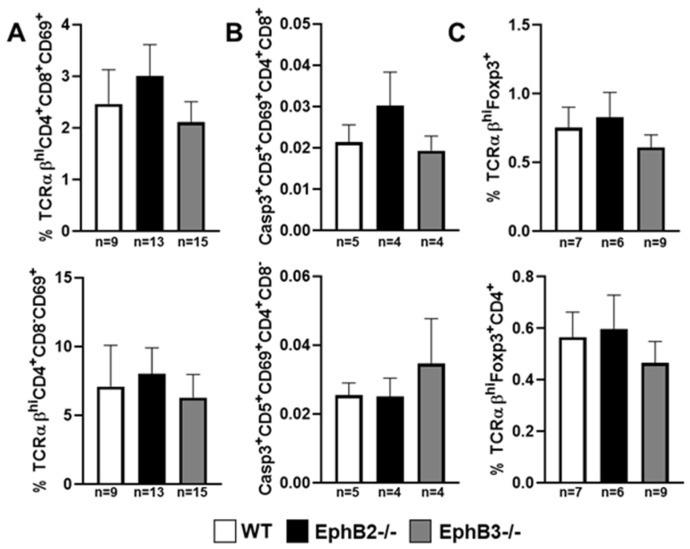
Percentages of positive and negative selected T-cells and T regulatory (Treg) cells in the thymus of EphB-deficient mice. Proportions of positive selected TCRαβ^hi^CD4^+^CD8^+^CD69^+^ and TCRαβ^hi^CD4^+^CD8^−^CD69^+^ thymocytes (**A**), negative selected Casp3^+^CD5^+^CD69^+^CD4^+^CD8^+^ cells and Casp3^+^CD5^+^CD69^+^CD4^+^CD8^−^ cells (**B**), and total TCRαβ^hi^Foxp3^+^ and TCRαβ^hi^Foxp3^+^CD4^+^ thymic Treg cells (**C**). Non-significant differences were found between WT and mutant values following the one-way ANOVA test with Tukey post hoc test. Caspase3 (Casp3). *n*, number of studied animals.

**Figure 3:**
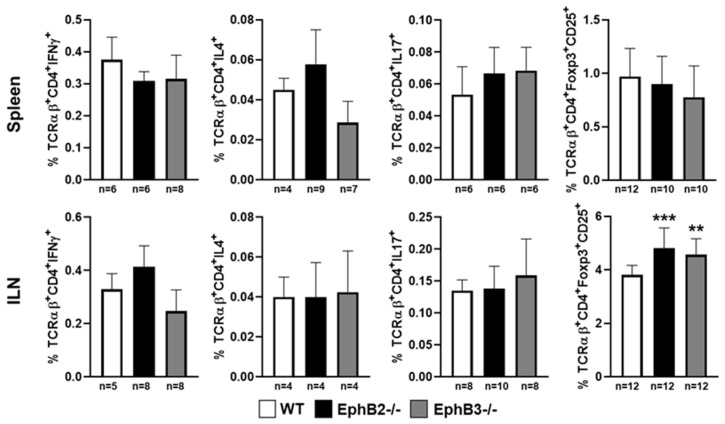
Proportions of Th1 (TCRαβ^+^CD4^+^IFNγ^+^), Th2 (TCRαβ^+^CD4^+^IL4^+^), Th17 (TCRαβ^+^CD4^+^IL17^+^) lymphocytes, and Treg cells (TCRαβ^+^CD4^+^CD25^+^FoxP3^+^) in both spleen and inguinal lymph nodes (ILN) in EphB-mutant mice. The significance of the one-way ANOVA test with Tukey post hoc test between WT and mutant values is indicated as: ** *p* < 0.01; *** *p* < 0.001. *n*, number of studied animals.

Despite the important alterations undergone by the epithelial component, the EphB-deficient thymi show little changes in the thymocyte phenotype. Although, as mentioned above, reduced percentages of lymphoid progenitor cells seed the mutant thymi, there are no significant changes in the percentages of distinct thymocyte subsets subsequently [98], except for a slight increase of DN1 (CD4^−^CD8^−^CD44^+^CD25^−^)cell proportions and reduced DN3 thymocytes [107,108], and increased proportions of Vβ3CD4^+^ cells that suggest certain altered thymocyte selection. On the contrary, there were no differences between mutant and WT thymuses in the proportions of neither positive selected thymocytes or negative selected cells, nor in the percentage of total thymic Treg cells. In addition, in the periphery no changes occur in the proportions of Th1, Th2, and Th17 [98], and the percentage of Treg remains unchanged in the spleen but increases slightly in mutant inguinal lymph nodes (ILNs), as compared with WT values [98]. These results have been recently confirmed (Figure 2 and Figure 3).

## 6. Does the Lack of Eph and/or Ephrins Affect the Thymic Selection?

At first glance, we might think that Eph and/or ephrins could directly affect the central tolerance. In this respect, the available data are very limited and contradictory. No changes in the T-cell maturation have been reported in mice with conditionally deleted EphB4 gene in TECs [122], deficiency in EphB6 [123] or in four Ephs together, EphB1, EphB2, EphB3, and EphB6 [124]. In addition, the deletion of ephrin-B1 or ephrin-B2 in thymocytes does not induce thymus phenotypes [125,126], but the absence of the two ephrins alters thymocytes and thymic structure [127] and induces low sensitivity to distinct autoimmune models [127,128]. We also reported reduced proportions of positive selected TCRαβ^hi^CD69^+^ thymocytes in some mice with ephrin-B1 and/or ephrin-B2 deleted in TECs [35] and EphA4-deficient thymuses show reduced percentages of both DP TCRαβ^hi^ cells and CD69^+^ cells [129].

Another possibility is to consider that EphB affects thymus phenotype via thymocyte-TEC interactions or through molecules involved in the generation of functional thymocytes. It is difficult to explain how the described alterations in thymic epithelial network do not impair, partially at least, the thymocyte development. Remarkably, altered thymic architecture has been associated with autoimmunity [130]. For example, individuals with Down’s syndrome have altered thymic histological organization and cell content and frequently develop autoimmune diseases by breakdown of central tolerance [131].

The so-called thymic nurse complexes (TNC), where single cTECs intimately interact with 5–30 thymocytes governing their secondary TCRα chain rearrangements, are a good experimental model to analyze the thymocyte–TEC interactions occurring in the thymic cortex [132]. Recently, we demonstrated that TNCs derived from EphB-deficient thymuses yielded fewer complexes due to the reduction of the numbers of TNCs containing 6–10 thymocytes, the most frequent in WT thymuses, but there were no changes in the proportions of positive selected TCRαβ^hi^CD69^+^ thymocytes [98]. In addition, the number and establishment of cell conjugates between TECs and DP thymocytes are altered when established with mutant DP cells [133].

These results are important because they question whether the observed defects in EphB mutant thymuses are really sufficient to impair the functional properties of thymocytes. A similar conclusion can be reached when considering the alterations in the expression of several molecules in the EphB-deficient thymuses. According to our results, the expression of numerous molecules involved in the functional interactions between TECs and thymocytes (i.e., Dll4, IL7/IL7R, β5t, Aire, RANK/RANKL, MHCII, CD80) are importantly reduced in mutant thymuses. Why do these changes not result in altered positive and/or negative selection of thymocytes? In other models, decreased Aire expression predisposes to autoimmunity [134,135].

Other possibilities are that either other cells implicated in thymocyte education, largely DCs, can partially reinforce the reduced activity of TECs or that just a few unaltered areas of thymic stroma are sufficient to support a fairly normal T-cell development [82,99,100].

As mentioned above, DCs participate in the elimination of self-reactive thymocytes and the formation of Treg cells by transporting peripheral TSA into the thymus [1,84,85] or after peripheral tissue antigens (PTA) transfer from mTECs [136,137,138]. Although we have no data on the percentages of DCs in EphB-deficient thymi, in EphA4^−/−^ mice, whose thymi show also profound epithelial alterations, there were no changes in DC proportions (Data not shown).

## 7. How Many TECs Are Necessary for Supporting a Proper T-Cell Maturation?

**Figure 4:**
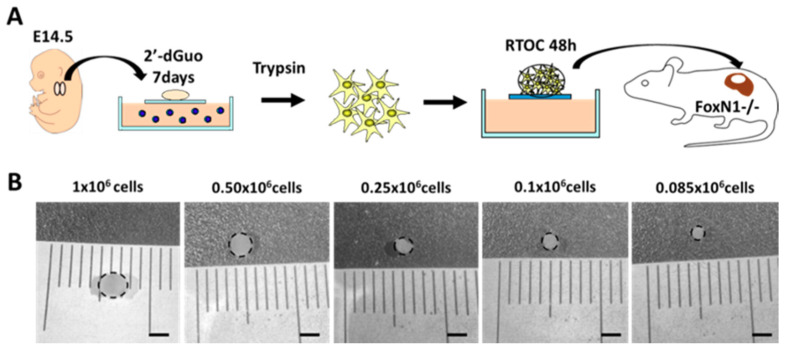
Diagram of reaggregate thymus organ culture (RTOC) establishment and representative images of the yielded thymi one month after grafting under the kidney capsule of FoxN1^−/−^ mice. (**A**) E14.5 WT thymic fetal lobes were treated with 2′-dGuo. After 7 days, thymic lobes containing just stromal cells were disaggregated with trypsin and reaggregated (RTOC) using different cell numbers (1, 0.5, 0.25, 0.1, and 0.085 × 10^6^ cells) and cultured for 48 h. Then, RTOCs were grafted under the kidney capsule of FoxN1^−/−^ mice for 1 month. (**B**) Representative RTOCs recovered one month after grafting. Notice the gradual reduction in the lobe size as compared with control RTOCs (1 × 10^6^ cells). Dotted line marks the limits of RTOC. Scale bar: 2 mm.

With respect to the possibility that a few thymic areas could be sufficient to ensure proper thymocyte maturation, we analyze the thymocyte differentiation in thymic cell reaggregates organized from different numbers of thymic stromal cells (TSC, 1–0.085 × 10^6^ cells) grafted under the kidney capsule of FoxN1^−/−^ mice. Reaggregates (RTOCs) had been established from E14.5 WT thymic lobes treated with 2′-dGuo for 7 days to eliminate thymocytes and, in general, any dividing thymic cells (Figure 4A). One month after grafting, thymic reaggregates as well as FoxN1^−/−^ spleen and ILNs were isolated and their lymphoid cell subsets examined by flow cytometry. Only those reaggregates that importantly grew after grafting were evaluated; nevertheless, the obtained results must be considered preliminary because of the low numbers of aggregates that we have been capable to analyze and some experiments have not been performed due to technical problems in our animal facilities.

Gross-anatomy of grafted one-month-old RTOCs confirmed that reaggregates established with higher numbers of thymic stromal cells produced both larger sized-thymic lobes (Figure 4B) and higher numbers of yielded thymic cells (Table 1). Thus, RTOCs established from 1–0.5 × 10^6^ stromal cells grew in vivo around 5–7 times, one month after grafting under the kidney capsule of FoxN1^−/−^ mice (Table 1). On the contrary, when RTOCs formed with lower numbers of stromal cells (0.25–0.085 × 10^6^ cells) were used, the number of yielded thymic cells was significantly lower (Table 1) and the observed growth one month later was around 3–4 times.

These variations in the amount of yielded thymic cells correlated well with changes in the proportions of the most numerous thymocyte subsets, DP cells and TCRαβ^hi^CD4^+^ cells (Table 1 and Table 2). Whereas RTOCs constituted with 1 × 10^6^ thymic stromal cells showed normal proportions of the distinct thymocyte subsets, quite similar to those reported in 2-month-old adult thymi [139], in RTOCs established with lower numbers of cells, there were some alterations in the T-cell maturation (although not statistically significant) consisting in gradual decreased frequencies of DP cells (Table 1) and increased percentages of TCRαβ^hi^ thymocytes, largely TCRαβ^hi^CD4^+^ T-cells (Table 2). This altered pattern of T-cell differentiation was particularly evident in grafted RTOCs formed with 0.085 × 10^6^ cells, in which the found values were statistically significant as compared to grafted RTOCs formed with 1 × 10^6^ stromal cells (Table 1 and Table 2). In periphery, preliminary results demonstrated a similar condition with reduced proportions of TCRαβ^hi^ T lymphocytes in both spleen and ILNs of FoxN1^−/−^ mice four months after to be grafted with RTOCs initially containing 0.1 × 10^6^ thymic stromal cells. In any experimental situation, FoxN1^−/−^ spleen or lymph nodes did not contain T lymphocytes one month after grafting, apart from those always present in non-grafted FoxN1^−/−^ peripheral lymphoid organs (Data not shown).

On the other hand, RTOCs containing the lowest numbers of thymic stromal cells showed significantly lower proportions of positively selected CD69^+^ cells within the TCRαβ^hi^ cell population that principally corresponded to the TCRαβ^hi^CD4^+^ cell subset (Table 3). However, the high percentage of the TCRαβ^hi^ cells occurring in these grafted RTOCs resulted in no significant differences with respect to the values observed in grafted RTOCs established with 1 × 10^6^ cells, when the total proportions of both thymic TCRαβ^hi^CD69^+^ cells and TCRαβ^hi^CD69^+^CD4^+^ cells were evaluated (Table 3).

A quite similar condition was observed when the proportion of Treg cells was examined in the distinct grafted RTOCs (Table 4). Remarkably, there were no significant differences in the proportions of Foxp3^+^ Treg cells into the TCRαβ^hi^ cell compartment between grafted RTOCs generated with distinct numbers of TSC. However, in those grafted RTOCs initiated with the lowest numbers of stromal cells (0.085 × 10^6^ cells), because they contain a high number of TCRαβ^hi^ cells, the proportions of both total TCRαβ^hi^Foxp3^+^ Treg cells and TCRαβ^hi^Foxp3^+^ CD4^+^ cells significantly increased with respect to the values observed in the other grafted RTOCs (Table 4). Unfortunately, we have currently no reliable data on the condition of negative selected thymocytes due to problems with the number of available embryonic mice.

In agreement with these results, previous studies using different experimental approaches determined a close relationship between the numbers of TECs and total thymocyte numbers [140,141]. These studies remarked also that, although the TEC numbers decreased 4 times, there were no changes in the proportions of distinct T-cell subsets. Thus, a low number of TECs would support normal T-cell differentiation, as also indicated in our current results in which a reduction of 10 or more times of thymic stromal cells in the initial RTOCs is necessary for observing significant alterations of the maturation of T-cell subpopulations. All together, these results confirm our hypothesis that a low number of TECs can be sufficient for a proper T-cell differentiation; only below that number it is impossible to have a normal T lymphopoiesis, but the proportions of both positively selected thymocytes and Treg cells appear to be normal. Functional studies in progress and determination of the proportions of negatively selected thymocytes will confirm or refute definitively the immunological significance of these preliminary results.

## Figures and Tables

**Table 1:** Cellularity and proportions of thymocyte subsets.

RTOC (×10^6^ TSC)	After Graftment (×10^6^ cells)	% CD4^+^ (CD4^+^CD8^−^)	% DP (CD4^+^CD8^+^)	% DN (CD4^−^CD8^−^)	% CD8^+^ (CD4^−^CD8^+^)
1	5.05 ± 2.71	7.00 ± 2.27	87.95 ± 2.26	2.11 ± 0.10	2.94 ± 0.21
0.5	3.54 ± 2.41	8.94 ± 0.68	87.56 ± 0.59	1.00 ± 0.43	2.51 ± 0.85
0.25	1.03 ± 0.70	10.24 ± 1.50	84.24 ± 2.17	2.08 ± 0.26	3.45 ± 0.41
0.1	0.34 ± 0.18 *	8.96 ± 3.59	86.24 ± 5.46	3.11 ± 2.23	1.70 ± 1.01
0.085	0.26 ± 0.23 *	16.44 ± 3.80 *	76.49 ± 4.11 *	2.36 ± 0.78	4.72 ± 1.81

Data show the number of cells and the proportions of thymocyte subsets according to CD4/CD8 expression in RTOCs established with different numbers of thymic stromal cells (1, 0.5, 0.25, 0.1, and 0.085 × 10^6^) after one month of engraftment under the kidney capsule of FoxN1^−/−^ mice. The significance of the one-way ANOVA test with Dunnett post hoc test comparing with control RTOC (1 × 10^6^ cells) is indicated as: * *p* < 0.05. Number of studied RTOCs, *n* = 3.

**Table 2:** Proportions of mature TCRαβ^hi^ thymocytes.

RTOC(×10^6^ TSC)	% of Total TCRαβ^hi^	% of Total TCRαβ^hi^CD4^+^	% of Total TCRαβ^hi^CD8^+^	% of Total TCRαβ^hi^CD4^+^CD8^+^
1	9.20 ± 3.09	5.51 ± 1.96	1.36 ± 0.37	2.11 ± 0.72
0.5	11.03 ± 1.01	7.05 ± 0.46	1.57 ± 0.07	2.30 ± 0.50
0.25	13.17 ± 0.93	8.38 ± 0.97	1.93 ± 0.54	2.38 ± 0.47
0.1	9.49 ± 3.83	5.73 ± 2.42	1.15 ± 0.66	1.94 ± 0.15
0.085	20.76 ± 3.53 **	14.00 ± 3.21 **	3.11 ± 0.67 *	3.31 ± 0.44

Proportions of total TCRαβ^hi^ cells and total TCRαβ^hi^CD4^+^, TCRαβ^hi^CD8^+^, and TCRαβ^hi^CD4^+^CD8^+^ cells yielded by the different RTOCs one month after grafting. The significance of the one-way ANOVA test with Dunnett post hoc test comparing with control RTOC (1 × 10^6^ cells) is indicated as: * *p* < 0.05; ** *p* < 0.01. Number of studied RTOCs, *n* = 3.

**Table 3:** Proportions of positive selected thymocytes.

RTOC (×10^6^ TSC)	% of CD69^+^ within TCRαβ^hi^ Cells	% of CD69^+^ within CD4^+^ Gated in TCRαβ^hi^ Cells	% of Total TCRαβ^hi^CD69^+^	% of Total TCRαβ^hi^CD69^+^CD4^+^
1	55.06 ± 4.98	59.19 ± 5.23	4.76 ± 1.51	3.13 ± 1.09
0.5	49.84 ± 8.53	53.23 ± 9.16	5.42 ± 0.02	3.62 ± 0.45
0.25	46.62 ± 4.69	50.22 ± 1.46	6.99 ± 2.23	4.85 ± 1.65
0.1	45.45 ± 8.40	47.05 ± 12.12	4.51 ± 1.11	2.92 ± 1.06
0.085	39.02 ± 3.75 *	37.72 ± 3.62 *	8.49 ± 2.73	5.48 ± 2.18

Data show the proportions of CD69^+^ cells within the total TCRαβ^hi^ cells and in the CD4^+^ cell population gated in TCRαβ^hi^ cells. Proportions of total TCRαβ^hi^CD69^+^ and TCRαβ^hi^CD69^+^CD4^+^ cells yielded by the different grafted RTOCs are also indicated. The significance of the one-way ANOVA test with Dunnett post hoc test comparing with control RTOC (1 × 10^6^ cells) is indicated as: *p < 0.05. Number of studied RTOCs, *n* = 3.

**Table 4:** Proportions of T regulatory cells (Treg).

RTOC (×10^6^ TSC)	% of Foxp3^+^ within TCRαβ^hi^ Cells	% of Foxp3^+^ within CD4^+^ Gated in TCRαβ^hi^ Cells	% of Total TCRαβ^hi^Foxp3^+^	% of Total TCRαβ^hi^Foxp3^+^CD4^+^
1	3.24 ± 0.12	3.63 ± 0.39	0.25 ± 0.07	0.18 ± 0.05
0.5	3.73 ± 1.29	4.47 ± 1.68	0.36 ± 0.15	0.28 ± 0.10
0.25	2.64 ± 0.58	3.04 ± 0.71	0.30 ± 0.04	0.25 ± 0.03
0.1	3.24 ± 0.23	3.64 ± 0.13	0.23 ± 0.01	0.15 ± 0.01
0.085	3.46 ± 1.52	4.06 ± 2.25	0.57 ± 0.15 *	0.44 ± 0.12 *

Data show the FoxP3 expression in the total TCRαβ^hi^ cells and in CD4^+^ cell subset gated in TCRαβ^hi^ cells. Proportions of total Treg (TCRαβ^hi^Foxp3^+^) and TCRαβ^hi^Foxp3^+^CD4^+^ cells are also indicated. The significance of the one-way ANOVA test with Dunnett post hoc test comparing with control RTOC (1 × 10^6^ cells) is indicated as: * *p* < 0.05. Number of studied RTOCs, *n* = 3.
